# The Landmark Series: Advances in Preoperative Mediastinal Lymph Node Staging for Non-small Cell Lung Cancer (NSCLC)

**DOI:** 10.1245/s10434-025-17008-2

**Published:** 2025-03-01

**Authors:** Alison S. Baskin, Katemanee Burapachaisri, Shreya Guha, Jeffrey B. Velotta

**Affiliations:** 1 Department of Surgery, University of California-San Francisco, San Francisco, CA USA; 2 University of California-San Francisco School of Medicine, San Francisco, CA USA; 3 California Northstate University College of Medicine, Elk Grove, CA USA; 4 Department of Thoracic Surgery, Kaiser Permanente Northern California, Oakland, CA USA

## Abstract

Accurate mediastinal staging is essential for determining the extent of lung cancer, predicting prognosis, and guiding treatment strategies. Current clinical guidelines recommend preoperative invasive mediastinal staging for most patients with potentially resectable lung cancer, as imaging modalities alone often lack sensitivity and specificity. Invasive mediastinal staging techniques are categorized into surgical (e.g., cervical mediastinoscopy, video-assisted thoracic surgery) and nonsurgical (i.e., minimally invasive) approaches. Although cervical mediastinoscopy has historically been the gold standard, minimally invasive techniques have gained prominence in recent decades. These approaches, including endobronchial ultrasound-guided transbronchial needle aspiration (EBUS-TBNA), endoscopic ultrasound-guided fine-needle aspiration (EUS-FNA), and combined EBUS/EUS, provide improved accuracy and reduced morbidity compared with traditional surgical methods. EBUS-TBNA facilitates access to a broad range of lymph node stations with real-time ultrasound guidance, while EUS-FNA complements EBUS by enabling transesophageal lymph node sampling. Together, these techniques enable a more comprehensive mediastinal staging. This review examines five key trials that explore the expanding role of endobronchial and endoscopic techniques in mediastinal staging for non-small cell lung cancer, demonstrating how these advancements have transformed the diagnostic landscape.

For patients with lung cancer, mediastinal staging is critical to determining the extent of disease, understanding the prognosis, and informing the subsequent management. Specifically, accurate mediastinal staging helps differentiate early stage from advanced lung cancer diagnoses, which impacts treatment decisions and outcomes.^1^ Surgically fit patients without evidence of mediastinal lymph node (LN) metastases are frequently recommended curative-intent surgery as the initial step in their treatment. In contrast, combination chemoradiotherapy ± immunotherapy is often the first-line treatment for patients with locally advanced stage and/or metastatic disease.^2–6^ Mediastinal LN staging begins with obtaining a contrast-enhanced chest computed tomography (CT), which is usually followed by a positron emission tomography (PET) scan and/or integrated PET/CT imaging. Given that these imaging modalities have limited sensitivity and specificity, current professional society recommendations agree all patients with potentially resectable lung cancer, except patients with negative imaging and small peripheral tumors at low-risk for nodal spread, should undergo invasive preoperative mediastinal staging.^1,7–12^ Overall, an estimated 68% of all surgically treated patients have indications for preoperative invasive mediastinal staging, the accuracy of which is imperative given outcomes significantly vary on the basis of the completeness of staging.^13,14^

## Summary of Invasive Staging Approaches

Invasive mediastinal LN staging is categorized into surgical and nonsurgical approaches. Surgical approaches include mediastinoscopy (cervical or extended), video-assisted thoracic surgery (VATS), and anterior mediastinotomy (i.e., the Chamberlain procedure). Despite falling out of favor with the availability of emerging endoscopic approaches, mediastinoscopy has long represented the gold standard for mediastinal LN evaluation. Traditional cervical mediastinoscopy allows for direct visualization and biopsy of paratracheal and subcarinal LN through a small suprasternal incision under general anesthesia. However, it is limited in accessing the inferior and posterior mediastinum, as well as the aortopulmonary window, which is better evaluated using VATS or the Chamberlain procedure.^1,8,15^ Although cervical mediastinoscopy is less invasive than other surgical staging approaches, it still carries procedural risk, with morbidity and mortality rates of 2% and 0.08%, respectively.^8,16^

Over the last several decades, approaches to invasive mediastinal staging have favored minimally invasive techniques given evidence that these new modalities are more accurate and reduce procedural risk. Nonsurgical, or minimally invasive approaches, center around bronchoscopy/endoscopy and include techniques such as endobronchial ultrasound (EBUS) with transbronchial needle aspiration (TBNA) or fine-needle aspiration (FNA) and endoscopic ultrasound (EUS)-guided FNA often used in combination with EBUS-TBNA. EBUS-TBNA uses an integrated curvilinear, ultrasound bronchoscope to provide visualization and facilitate transbronchial sampling of LN under real-time ultrasonographic guidance, typically with a 22-gauge needle.^17^ This contrasts with blind TBNA, which is rarely used today. EBUS provides access to a wider range of LN stations, including the more distal posterior subcarinal and hilar regions, often beyond the reach of standard mediastinoscopy.^1^ Recently, EBUS-TBNA has been complemented by EUS-FNA, which places an ultrasound into the esophagus, allowing for transesophageal LN sampling with reduced morbidity compared with invasive surgical approaches.^18,19^ Together, EBUS-TBNA and EUS-FNA provide accessibility to all mediastinal LN stations besides the para-aortic nodes.

This review highlights several key trials examining endobronchial and endoscopic techniques for surgical mediastinal staging in non-small cell lung cancer (NSCLC), collectively supporting adoption of these contemporary approaches (Table 1).

**Table 1:** List of reviewed landmark studies

	Author (publication year)	Mediastinal lymph node staging modality examined
1	Herth et al. (2006)	EBUS-TBNA
2	Wallace et al. (2008)	TBNA versus EBUS versus EUS versus EBUS/EUS
3	Annema et al. (2010)	Surgical staging (i.e., mediastinoscopy) versus EBUS/EUS plus surgical staging
4	Um et al. (2015)	EBUS versus mediastinoscopy under conscious sedation
5	Bousema et al. (2023)	EBUS/EUS ± confirmatory mediastinoscopy

## STUDY 1: “Endobronchial Ultrasound-Guided Transbronchial Needle Aspiration of Lymph Nodes in the Radiologically Normal Mediastinum”

### Purpose and Rationale

In their 2006 study, Herth et al. investigated the accuracy of using EBUS-TBNA to stage patients with NSCLC with normal mediastinal LN on CT imaging.^20^ Prior to this work, EBUS had only been used to sample radiologically enlarged LN.

### Study Design and Endpoints

Patients with NSCLC without CT-enlarged mediastinal LN underwent EBUS-TBNA followed by surgical staging within 10 days. All visualized LN between 5 mm and 10 mm were aspirated, while normal-sized LN (< 5 mm) were not. The primary endpoint was the diagnostic accuracy of EBUS-TBNA in sampling LN < 1 cm in diameter.

### Results

Between 2003 and 2005, 100 patients underwent EBUS-TBNA and surgical staging, either via mediastinoscopy (15%) or thoracotomy with LN dissection (85%), to confirm aspiration findings. Despite negative CT scans, EBUS-TBNA detected malignancy in 19 patients. Surgical staging identified two additional patients with nodal metastases. EBUS accurately classified all 79 patients without N1/N2 disease. Overall, EBUS demonstrated a sensitivity of 92%, specificity of 100%, and negative predictive value (NPV) of 96%.

### Conclusions

EBUS-TBNA can identify nodal metastases in patients with radiologically normal mediastinal LN.

### Commentary

Herth et al. presented one of the first studies supporting the use of EBUS-TBNA for identifying nodal metastases in patients without CT-enlarged mediastinal LN. Notably, at the time of this study, PET was not routinely performed in practice. Although over the last two decades PET has become an important tool in non-invasive mediastinal staging, demonstrating higher sensitivity and specificity than CT, the findings of this study remain relevant.^7,11^ Specifically, through this work, Herth et al. influenced many subsequent studies comparing endoscopic staging techniques with more invasive approaches.

## STUDY 2: “Minimally Invasive Endoscopic Staging of Suspected Lung Cancer”

### Purpose and Rationale

With the rise of minimally invasive approaches to staging, Wallace et al. conducted this 2008 study to compare the diagnostic accuracy of traditional TBNA, EBUS-FNA, and the combination of EBUS-FNA and EUS-FNA (EBUS/EUS).^15^

### Study Methods and Endpoints

Patients with known or radiologically suspected non-metastatic lung cancer underwent TBNA, EUS, and EBUS performed sequentially as a single combined procedure. The primary endpoint was the sensitivity for detecting mediastinal LN metastases. Pathologic confirmation and 6- to 12-month clinical follow-up were the criterion standards.

### Results

A total of 138 patients were enrolled between 2004 and 2006, 30% of whom were found to have nodal metastases. Overall, EBUS and EUS were each more sensitive than standard TBNA in detecting malignant LN (69% vs. 69% vs. 36%) and had higher NPVs (88% vs. 88% vs. 78%). Combined EBUS/EUS had the highest sensitivity (93%) and NPV (97%) of any individual method or other paired combination. Furthermore, EBUS/EUS had the highest sensitivity and NPV for detecting LN in any mediastinal location and in patients with radiologically normal nodes.

### Conclusions

Combination EBUS/EUS was more diagnostically accurate and complete than TBNA, EUS, and EBUS-alone and was performed without procedural complications.


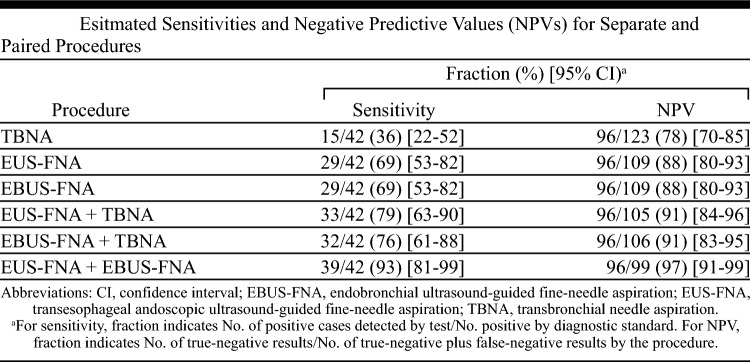


### Commentary

In contrast to prior work comparing the diagnostic accuracies of TBNA, EUS, and EBUS, this study provided a unique opportunity to perform paired comparisons of these procedures.^17,20–22^ Wallace et al. demonstrated that combined EBUS/EUS was superior to any procedure alone, even in subgroup analysis by LN station and in patients without mediastinal adenopathy. As they point out, this is likely because EBUS does not share the same “blind spot” of EUS, which is located immediately anterior to the trachea.^21^ With EUS alone, the anterior mediastinum is not visualized because the esophageal-placed ultrasound cannot penetrate the air-filled trachea. However, as demonstrated, by combining these two modalities, near-complete staging can be achieved given their complementary access to the anterior and posterior mediastinum.^21^ These findings are some of the first to suggest EBUS/EUS may be a viable alternative to surgical staging.

## STUDY 3: “Assessment of Surgical Staging versus Endoscopic Ultrasound (ASTER) Trial”

### Purpose and Rationale

In this 2010 study, Annema et al. initially planned to compare surgical staging (mediastinoscopy) with combined EBUS/EUS.^23^ However, given guidelines recommending confirmatory mediastinoscopy after negative endosonography, their primary analysis was changed to compare surgical staging alone with endosonography followed by surgical staging.

### Study Design and Endpoints

Patients with known or suspected NSCLC were assigned to surgical staging with mediastinoscopy alone or endosonography with combined EBUS/EUS. In patients without evidence of nodal metastasis on surgical staging or endosonography, lung resection with mediastinal lymph node dissection was performed via a thoracotomy, which was used as the reference standard. If nodal disease was identified, a thoracotomy was not performed. The primary endpoint was sensitivity for detecting mediastinal (N2/N3) nodal metastases. Secondary outcomes included rates of unnecessary thoracotomy and procedural complications.

### Results

From 2007 to 2009, a total of 241 patients were randomized to surgical staging (*n* = 118) or endosonography (*n* = 123). Among the patients in the endosonography group, 65 had no evidence of locally advanced disease and therefore also underwent surgical staging. This combined approach demonstrated significantly higher sensitivity compared with surgical staging alone (94% vs. 79%, *p* = 0.02), which consequently resulted in a significantly lower rate of unnecessary thoracotomies (7% vs. 18%, *p* = 0.02) compared with surgical staging alone. There were no significant differences in complication rates.

Additionally, the sensitivity of endosonography alone was similar to that of mediastinoscopy alone (85% vs. 79%, *p* = 0.47), however there were significantly fewer complications (1% vs. 6%, *p* = 0.03) associated with endosonography.


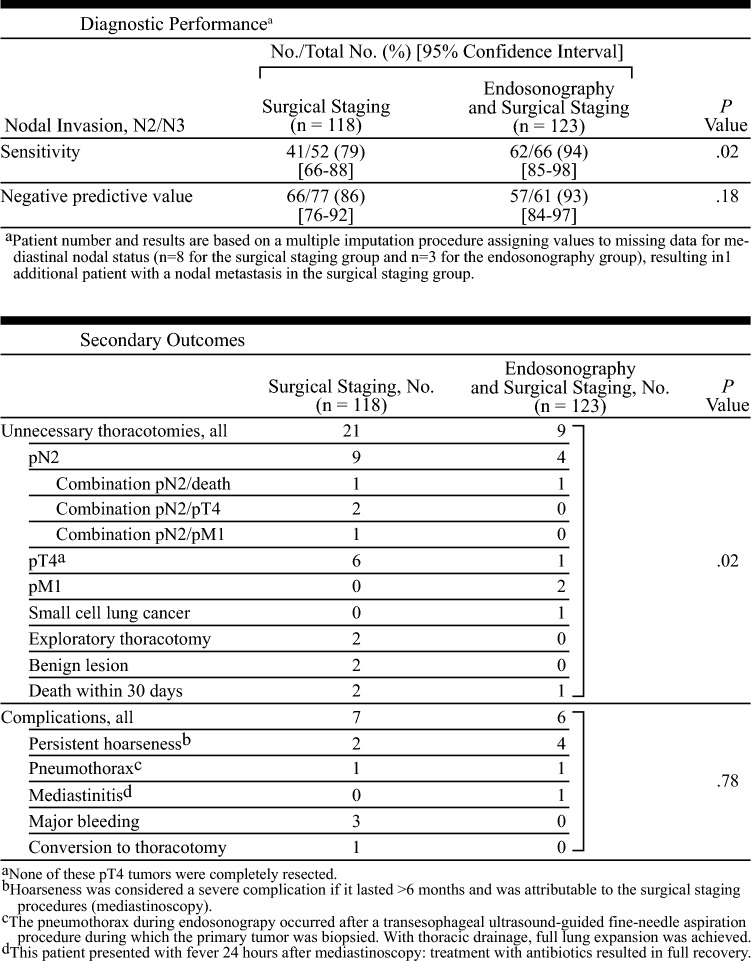


### Conclusions

Among patients with suspected NSCLC, staging with endosonography—followed by mediastinoscopy if negative—had greater sensitivity than mediastinoscopy alone and was associated with fewer unnecessary thoracotomies compared with surgical staging alone.

### Commentary

This randomized controlled multicenter study demonstrated that performing endosonography prior to surgical staging improves the detection of nodal metastases and reduces unnecessary thoracotomies compared to surgical staging alone. Interestingly, a post hoc survival analysis demonstrated no difference between the two groups at 5 years, which could be because the study was powered to detect sensitivity, not survival.^24^

The authors also found improved sensitivity for detection of nodal metastases when adding mediastinoscopy to a negative endosonography (85% to 94%), however, these data indicated 11 patients would need to undergo mediastinoscopy after a negative endosonography to identify a single patient with nodal metastases. These findings challenged the utility of performing routine mediastinoscopy after negative endosonographic and gave rise to subsequent work evaluating the need for confirmatory mediastinoscopy.^25^ Furthermore, a separate study assessing clinical and resource-use data from the ASTER trial found endosonography was associated with lower costs due to the reduced number of thoracotomies, as well as a better quality of life for patients undergoing staging.^26^

## STUDY 4: “Endobronchial Ultrasound versus Mediastinoscopy for Mediastinal Nodal Staging of Non-small-Cell Lung Cancer”

### Purpose and Rationale

Although a key advantage of EBUS is its ability to be performed under conscious sedation, at the time of this study, the only two prospective trials comparing EBUS with mediastinoscopy were designed with EBUS performed under general anesthesia.^27,28^ To evaluate the benefit of EBUS in a more routine clinical setting, Um et al. assessed the diagnostic performance of EBUS under conscious sedation in their 2015 study.^29^

### Study Design and Endpoints

Patients with NSCLC and suspicion of N1-N3 nodal metastases (≥ 1 cm LN and/or nodal 18F-fluorodeoxyglucose (FDG) uptake on PET/CT scan) underwent EBUS-TBNA followed by traditional cervical mediastinoscopy or video-assisted mediastinoscopy (VAM). The primary endpoint was to compare the sensitivity of EBUS with that of mediastinoscopy in detecting N2/N3 metastases. Secondary endpoints included comparisons of specificity, accuracy, positive predictive value (PPV), and NPV. The diagnostic standards were cytopathologic confirmation using all available tissue-sampling methods (i.e., EBUS, mediastinoscopy, or surgical LN dissection).

### Results

Between 2010 and 2012, a total of 127 patients underwent both EBUS and mediastinoscopy. N2/N3 metastases were confirmed in 59% of patients. On a per-person analysis, EBUS demonstrated significantly greater diagnostic sensitivity (88% vs. 81%), accuracy (93% vs. 89%), PPV (100% vs. 89%), and NPV (85% vs. 79%) compared with mediastinoscopy (all *p* < 0.005). Specificity was 100% for both procedures. In a subgroup analysis by nodal station, EBUS was significantly more sensitive than mediastinoscopy at paratracheal station 4L (81 vs. 52%, *p* = 0.027).


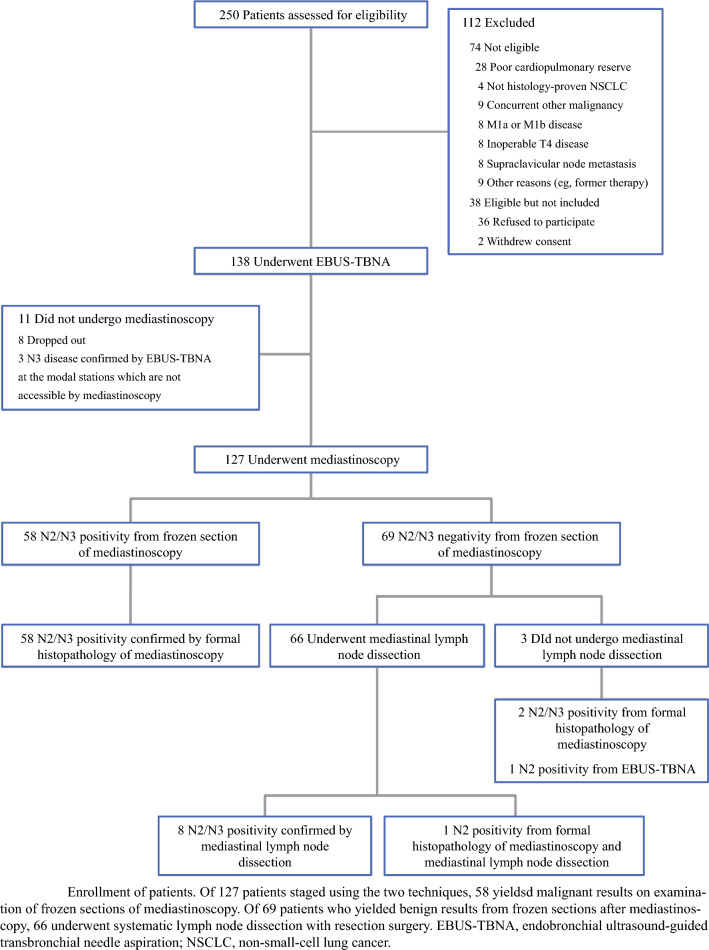


### Conclusions

In this single-center prospective study, EBUS performed under conscious sedation had better diagnostic sensitivity, accuracy, and NPV for mediastinal staging of cN1-3 NSCLC.

### Commentary

This trial provided new evidence supporting the clinical utility of EBUS as a more effective approach to mediastinal staging than cervical mediastinoscopy in patients with radiologically enlarged or FDG-avid paratracheal and subcarinal LN. Prior work had shown EBUS performs as well, if not better than, mediastinoscopy across nearly all metrics.^27,28^ However, in both prior studies, EBUS was performed concurrently with mediastinoscopy under general anesthesia, raising concerns abouts its practicality in routine clinical settings. In this trial, Um et al. not only demonstrated that EBUS is superior to mediastinoscopy under conscious sedation, but also emphasized its advantage in sampling LN located near delicate structures (e.g., 4L, which is located near the ascending aorta, pulmonary artery, and recurrent laryngeal nerve) or deep within the chest. Although this study did not evaluate patients with enlarged pretracheal nodes—a region where mediastinoscopy is typically preferred—it provided compelling evidence supporting EBUS as the first-line procedure for mediastinal staging.^30^

## STUDY 5: “Endosonography With or Without Confirmatory Mediastinoscopy for Resectable Lung Cancer: A Randomized Clinical Trial” (MEDIASTrial)

### Purpose and Rationale

Guidelines recommend confirmatory mediastinoscopy for patients with NSCLC and a high probability of mediastinal involvement, even after negative endosonographic staging. However, the necessity of confirmatory mediastinoscopy has been a topic of debate. Given a lack of randomized clinical data, Bousema et al. conducted this randomized controlled noninferiority study to evaluate whether confirmatory mediastinoscopy is necessary following negative endosonographic staging.^25^

### Study Design and Endpoints

Patients with proven or suspected NSCLC and an indication for mediastinal staging after negative systemic EBUS with or without EUS (i.e., cN1-3 disease or large, central, or FDG-non-avid tumors) were enrolled. Following negative endosonography, patients were assigned 1:1 to undergo immediate lung tumor resection with LN dissection (immediate resection group) or confirmatory cervical VAM followed by tumor resection (mediastinoscopy group). The primary endpoint was the presence of unforeseen N2 disease (uN2) after tumor resection with LN dissection. The noninferiority margin was 8% (*P*_noninferior_ < 0.025).^23,31^ Secondary endpoints included time-to-treatment and adverse events.

### Results

A total of 360 patients with negative endosonography were enrolled from 2017 to 2020; 178 patients were assigned to immediate resection and 182 to confirmatory mediastinoscopy. The mean interval between endosonography and tumor resection was 28 days in the immediate resection group and 38 days in the mediastinoscopy group. Mediastinoscopy detected metastases in 8.0% of patients. The uN2 rate after immediate resection (8.8%) was noninferior compared with mediastinoscopy first (7.7%) in both intention-to-treat (*P*_noninferior_ = 0.014) and per-protocol (*P*_noninferior_ = 0.016) analyses. Major morbidity and 30-day mortality was higher in the mediastinoscopy group, but this difference was not statistically significant.


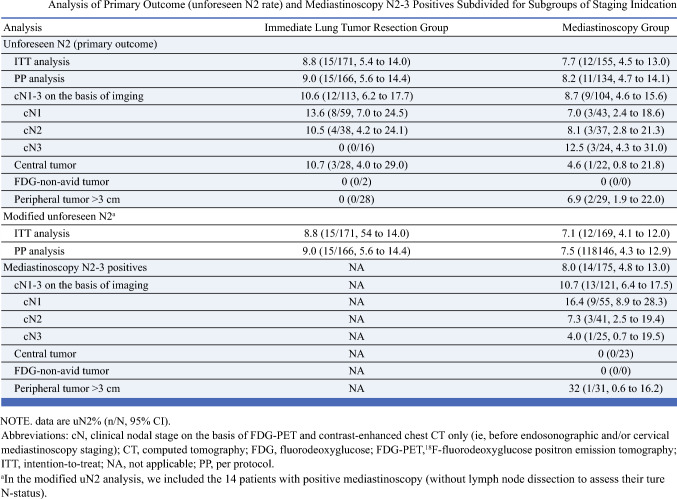


### Conclusions

EBUS alone is noninferior to EBUS with confirmatory mediastinoscopy in patients with resectable NSCLC.

### Commentary

Using survival data from the ASTER trial and their own systematic review to define the noninferiority limits, Bousema et al. demonstrated confirmatory mediastinoscopy after negative systematic endosonography may be safely omitted in select patients.^14,23^ In their editorial on the MEDIASTrial, Farjah et al. discuss several of the study’s limitations including the lower prevalence of nodal disease in the resection group compared with the mediastinoscopy group, the study’s much lower overall prevalence of nodal disease compared with the ASTER trial, and the applicability of the findings in an era of new multimodality treatment options.^32^ Despite these limitations, this study is acknowledged for offering valuable insights into the tradeoffs between invasive staging strategies, including staging accuracy, treatment selection, procedural risk, and time-to-treatment initiation.^32^ Future research should aim to identify the specific patients in whom mediastinoscopy is necessary following negative endosonography, as well as evaluate how the need for adequate tissue samples for biomarker testing might influence preoperative staging strategies.^33^

## Current Society Guidelines

Recognizing the safety and efficacy of these minimally invasive techniques, current society recommendations favor EBUS/EUS as the first-line procedure for mediastinal staging. Specifically, the European Society of Thoracic Surgeons (ESTS) guidelines, revised in 2014, recommend EBUS/EUS with FNA as first choice in cases of enlarged mediastinal LN on CT or PET-positive LN.^9^ The 2024 National Comprehensive Cancer Network (NCCN) clinical guidelines agree, suggesting patients undergo the least invasive biopsy with the highest yield as the first diagnostic study.^10^ Furthermore, the American College of Chest Physicians (ACCP) recommends performing a needle-based technique (e.g., EBUS, EUS, or EBUS/EUS) as the best first step over surgical staging in patients with and without discrete nodal findings on CT or PET.


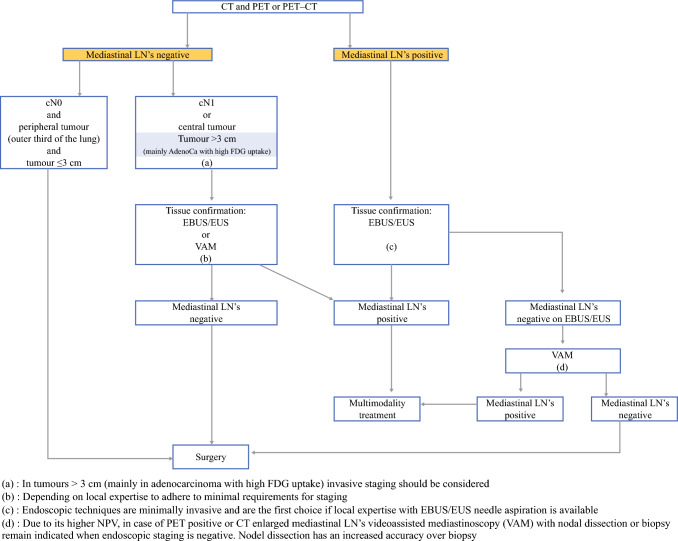


## Future Directions

With recent advances in robotics, navigational bronchoscopy, and artificial intelligence (AI), studies are now aiming to better understand the potential role of these newer modalities in mediastinal LN staging and lung nodule biopsy. Currently some of the studies actively recruiting participants include:

- “The SEQUENCE Trial: Evaluating Diagnostic Yield of Robotic-assisted Bronchoscopy When Staging EBUS is Performed First or Second in the Same Procedure”: a multicenter randomized clinical trial aiming to determine whether sequence of staging EBUS plays a role in diagnostic yield, incidence of atelectasis, and safety outcomes in patients undergoing robotic-assisted bronchoscopy.^34^
- “Augmented Endobronchial Ultrasound (EBUS-TBNA) with Artificial Intelligence”: a multicenter prospective feasibility study exploring how a machine learning algorithm may be used to identify LN and blood vessels examined with EBUS.^35^
- “Navigation Endobronchial Ultrasound (NEBULA)”: a non-blinded randomized controlled trial aiming to determine whether a combination or robotic-EBUS and electromagnetic navigation bronchoscopy (ENB) is superior to ENB alone in biopsy sampling of lung nodules.^36^

## Conclusions

Over the last two decades, studies have supported a shift toward less invasive mediastinal LN staging, particularly with the advent of bronchoscopic and endoscopic techniques. As illustrated by the studies in this Landmark Series, combined EBUS/EUS and even EBUS alone is a superior alternative to cervical mediastinoscopy, especially given their reduced risk of procedural complications. Furthermore, there is evidence that confirmatory mediastinoscopy may be omitted in select patients who have had negative endoscopic staging.

Realistically, combined EBUS/EUS is not feasible at many institutions given the need for specialized equipment and combined endoscopic and bronchoscopic expertise and availability.^15^ EUS is typically performed by interventional gastroenterology, while either thoracic surgery or pulmonology performs EBUS, meaning coordination between multiple specialists is often necessary. Accordingly, current guidelines recognize this as a limiting factor, which, along with many considerations, may influence how mediastinal staging is performed. Furthermore, as highlighted by many of these landmark studies, the accuracy and completeness of EBUS and EUS depend heavily on the experience and skill of the proceduralist, which may limit their reproducibility across all clinical settings. Thus, in addition to continuing to explore novel techniques to mediastinal LN staging, future efforts may be focused on increasing and improving access to high-quality minimally invasive staging through expanded training and education, availability of staging equipment and technologies, and additional support for multispecialty collaboration.
